# New York Society for the Relief of the Widows and Orphans of Medical Men

**Published:** 1842-06-04

**Authors:** 


*New York Society for the relief of the Widows and Orphans of Medical Men.* A meeting of the Profession in New York was held on the 14th of May, Dr. Mott in the chair, at which an organization was adopted for the above purpose. The N. Y. Medical Gazette states that subscriptions to the new society have been received in considerable numbers.

